# Quantifying alcohol’s harm to others: a research and policy proposal

**DOI:** 10.2471/BLT.24.291338

**Published:** 2024-04-30

**Authors:** Carolin Kilian, Jakob Manthey, Charlotte Probst

**Affiliations:** a Institute for Mental Health Policy Research, Centre for Addiction and Mental Health (CAMH), 33 Ursula Franklin Street, Toronto, Ontario, M5S 2S1, Canada.; b Department of Psychiatry, University Medical Center Hamburg-Eppendorf, Hamburg, Germany.

## Abstract

Just under 2.5 million people die annually due to alcohol use. This global estimate, however, excludes most of the health burden borne by others than the alcohol user. Alcohol’s harm to others includes a multitude of conditions, such as trauma from traffic crashes, fetal disorders due to prenatal exposure to alcohol, as well as interpersonal and intimate partner violence. While alcohol’s causal role in these conditions is well-established, alcohol’s harm to others’ contribution to the overall health burden of alcohol remains unknown. This knowledge gap leads to a situation in which alcohol policy and prevention strategies largely focus on the reduction of alcohol’s detrimental health harms on the alcohol users, neglecting affected others and population groups most vulnerable to these harms, including women and children. In this article, we seek to elucidate why estimates for alcohol’s harm to others are lacking and offer guidance for future research. We also argue that a full assessment of the alcohol health burden that includes the harm caused by others’ alcohol use would enhance the visibility and public awareness of such harms, and advancing the evaluation of policy interventions to mitigate them.

## Résumé

Chaque année, un peu moins de 2,5 millions de décès sont liés à la consommation d'alcool. Cette estimation globale ne tient cependant pas compte de l'impact sur la santé de l'entourage des consommateurs d'alcool. Les méfaits de l'alcool sur les autres ont une multitude de conséquences, parmi lesquelles des traumatismes dus aux accidents de la circulation, des anomalies fœtales liées à une exposition prénatale à l'alcool, ainsi que des actes de violence interpersonnelle et entre partenaires. Bien que le rôle causal de l'alcool dans ces problématiques soit bien établi, les répercussions de tels méfaits sur la santé dans son ensemble restent à déterminer. Des lacunes qui aboutissent souvent à une situation dans laquelle les politiques et stratégies de prévention se concentrent principalement sur la diminution des effets néfastes de l'alcool sur la santé des consommateurs eux-mêmes, négligeant les personnes qui les entourent et les catégories de population les plus vulnérables, en particulier les femmes et les enfants. Dans cet article, nous tentons d'expliquer pourquoi il n'existe aucune estimation concernant les méfaits de l'alcool sur les autres et prodiguons des conseils pour de futures recherches. Nous plaidons aussi pour une analyse complète de la charge sanitaire imputable à l'alcool incluant les méfaits de l'alcool sur les autres, afin d'améliorer la visibilité et de mieux sensibiliser l'opinion publique à ces problématiques, mais aussi de faire progresser l'évaluation des interventions politiques entreprises pour y remédier.

## Resumen

Cerca de 2,5 millones de personas mueren cada año por el consumo de alcohol. Sin embargo, esta estimación global excluye la mayor parte de la carga sanitaria que soportan personas que no son consumidores de alcohol. Los daños del alcohol a terceros incluyen multitud de afecciones, como los traumatismos por accidentes de tráfico, los trastornos fetales debidos a la exposición prenatal al alcohol, y la violencia interpersonal y de pareja. Aunque se sabe que el alcohol influye en estas afecciones, se desconoce la contribución de los daños del alcohol a terceros a la carga sanitaria global que supone el alcohol. Esta falta de conocimiento conduce a una situación en la que las estrategias de política y de prevención del alcohol se centran en gran medida en la reducción de los daños perjudiciales del alcohol para la salud de los consumidores de alcohol, dejando de lado a los demás afectados y a los grupos de población más vulnerables a estos daños, incluidas las mujeres y los niños. En este artículo, tratamos de dilucidar por qué faltan estimaciones sobre los daños del alcohol en otras personas y ofrecemos orientaciones para futuras investigaciones. También argumentamos que una evaluación completa de la carga sanitaria del alcohol que incluya los daños causados por el consumo de alcohol de otras personas mejoraría la visibilidad y la concienciación pública de esos daños, y haría avanzar la evaluación de las intervenciones políticas para mitigarlos.

## ملخص

يموت أقل قليلًا من 2.5 مليون شخص سنويًا بسبب تعاطي الكحول. ومع ذلك، فإن هذا التقدير العالمي يستثني معظم العبء الصحي الذي يتحمله أشخاص آخرون لا يتعاطون الكحول. إن ضرر الكحول للآخرين يتضمن عددًا كبيرًا من الحالات، مثل الإصابات من حوادث المرور، اضطرابات الجنين بسبب التعرض للكحول قبل الولادة، فضلاً عن العنف بين الأشخاص، والعنف بين الشركاء الحميمين. على الرغم من أن الدور السببي للكحول في هذه الحالات متأصل وثابت، إلا أن مساهمة أضرار الكحول في الأعباء الصحية العامة للكحول، لا تزال غير معروفة. وتؤدي هذه الفجوة في المعرفة إلى موقف تركز فيه سياسات الكحول واستراتيجيات الوقاية، بشكل كبير، على الحد من الأضرار الصحية الضارة للكحول على متعاطي الكحول، وإهمال الآخرين المتضررين، والفئات السكانية الأكثر عرضة لهذه الأضرار، بما في ذلك النساء والأطفال. نسعى في هذه المقالة إلى توضيح سبب الافتقار لوجود تقديرات لأضرار الكحول على الآخرين، وتقديم توجيهات للبحث المستقبلي. كما نرى أن التقييم الكامل للعبء الصحي للكحول، الذي يشمل الضرر الناجم عن تعاطي الآخرين للكحول، سوف يعزز الرؤية والوعي العام بهذه الأضرار، والارتقاء بتقييم التدخلات السياسية للتخفيف منها.

## 摘要

每年有将近 250 万人死于饮酒。但是，该全球估计值未考虑除饮酒者以外的其他人承受的大部分健康负担。酒精对他人造成的伤害包含多种情况，例如因例如因交通事故所造成的伤害、产前饮酒导致的胎儿缺陷、交通事故以及人际和亲密伴侣暴力。虽然我们可以确认酒精是导致这些情况出现的原因所在，但因酒精导致的整体健康负担加重而对他人造成的伤害仍未可知。由于存在这种认识差距，导致酒精政策和预防策略主要侧重于减少酒精对饮酒者造成的有害健康伤害，而忽视了受影响的其他人以及最容易受到此类伤害影响的人群，包括妇女和儿童。在本文中，我们试图阐明未能就酒精对他人造成的伤害进行估算的原因，并为未来的研究提供指导。我们还认为，对酒精健康负担（包括因他人饮酒而造成的伤害）进行全面评估，有利于提高此类伤害的可见性以及公众对此类伤害的认识，并推进对为减轻这些伤害而实施的政策干预措施的评估。

## Резюме

Ежегодно из-за употребления алкоголя умирает почти 2,5 миллиона человек. Однако эта глобальная оценка не учитывает большую часть воздействия на здоровье людей, не употребляющих алкоголя. Вред от употребления алкоголя для окружающих включает в себя множество проявлений, таких как травмы, полученные в результате дорожно-транспортных происшествий, нарушения развития плода из-за пренатального воздействия алкоголя, а также межличностное насилие и насилие над сексуальным партнером. Хотя причинная роль алкоголя в развитии этих случаев общеизвестна, вклад алкоголя в общее бремя алкоголизма для здоровья окружающих остается неизвестным. Этот пробел в знаниях приводит к тому, что политика и стратегии профилактики в отношении алкоголя в основном сосредоточены на снижении пагубного воздействия алкоголя на здоровье людей, употребляющих алкоголь, и не учитывают интересы других людей и групп населения, наиболее уязвимых к этому вреду, включая женщин и детей. В этой статье рассматриваются причины отсутствия оценок вреда, полученного окружающими вследствие употребления алкоголя другими людьми, и предлагаются рекомендации для будущих исследований. Кроме того, существует мнение, что полная оценка бремени алкоголизма, включающая вред, причиняемый употреблением алкоголя другими людьми, позволит повысить уровень осведомленности общественности о таком вреде и улучшить оценку политических мер, направленных на его смягчение.

## Introduction

Just under 2.5 million people die annually due to alcohol use.^1^ This global estimate, however, excludes most of the health burden borne by others than the alcohol user. This so-called alcohol’s harm to others encompasses a broad spectrum of conditions, including financial, emotional, physical and sexual harms affecting families, workplaces and communities.^2^ Available data suggest a significant scope of alcohol’s harm to others. For example, a modelling study estimated that about 119 000 children are born with fetal alcohol syndrome per year.^3^ A study addressing interpersonal violence in the Global Burden of Disease (GBD) regions of High Income and Central Europe, Eastern Europe and Central Asia estimated that in 2019, approximately one in five and one in 20 adults had in the past year experienced emotional and physical violence from others’ alcohol use, respectively.^4^ While alcohol’s causal role in these conditions is inherent by definition, alcohol’s harm to others’ contribution to the overall health burden of alcohol remains unknown. This knowledge gap leads to a situation in which alcohol policy and prevention strategies largely focus on the reduction of alcohol’s detrimental health harms on the alcohol users, neglecting not only affected others but also population groups most vulnerable to these harms, including women and children.

In tobacco control policy, research evidence on the harmful effects of second-hand smoking brought a considerable shift in national policy strategies. Nowadays, tobacco control policies are driven by the aim of preventing health risks in both smokers and non-smokers through establishing smoke-free environments, among other initiatives.^5^ However, although alcohol has been demonstrated to cause more harm to others than any other psychoactive substance from a list of 20 legal and illegal drugs,^6^ such policy arguments appear to be largely absent for alcohol. We argue that this situation reflects the lack of burden of disease estimates for alcohol’s harm to others, which conceals their consequences and prevents their adequate consideration in policy debates. Including alcohol’s harm to others in the burden of disease framework will allow for an evidence-based assessment of the entire health burden caused by alcohol, thereby enhancing its visibility and public awareness, and advancing the evaluation of policy interventions to mitigate these harms.

## Determining the health burden 

The question arises as to why alcohol’s harm to others is insufficiently captured in burden of disease analysis – a critical shortcoming previously stressed in a 2019 World Health Organization (WHO) report on this issue.^2^ Little progress has been made since the publication of this report and, to the best of our knowledge, only two studies to date quantify alcohol’s harm to others’ burden of disease (in Germany^7^ and New Zealand^8^). In this article, we seek to elucidate why such estimates for alcohol’s harm to others are lacking and offer guidance for future research. To this end, we use the example of interpersonal violence, which is among the leading causes of deaths among 15-to-49-year-olds^9^ and has a strong causal link to alcohol use.

Two general approaches to determine the health burden to others exist – a direct and an indirect approach. The direct approach requires dyadic data, that is, information on the health outcome of interest (for example, interpersonal violence) while at the same time ascertaining information on alcohol use of the persons involved. Doing so allows researchers to determine the share of events in which the exposure has causally contributed to the outcome. For example, in a study, patients with violent injuries were interviewed in emergency departments of 14 countries on whether they believed the person(s) who inflicted the injury had been drinking alcohol, and whether the incident would have happened without alcohol – that is, causal attribution.^10^ Their results suggest that an estimated 15% of violent injuries could be attributed to the alcohol use of another person. In other words, these injuries would not have occurred without others’ alcohol use.

The example illustrates the major challenges of the direct approach. First, a dyadic database is required; second, causality between the alcohol use of one person and the outcome in another person needs to be established. The latter information is usually not available from routine statistics such as hospital or death registries, which is why surveys remain the most commonly used direct source for studying alcohol’s harm to others. However, the validity of such surveys is questionable, given their well-established limitations, including self-reporting biases, inconsistencies in assessment and decreasing response rates.^11^ Moreover, subjective evaluations of causality may differ across societies, given sociocultural differences in the willingness to make attributions of alcohol’s involvement in harm, and differences in thresholds of perceived harm.^12^ Police records can provide additional information on recorded crimes involving alcohol use; however, alcohol involvement may not be routinely assessed.

## A research agenda 

**Fig. 1:**
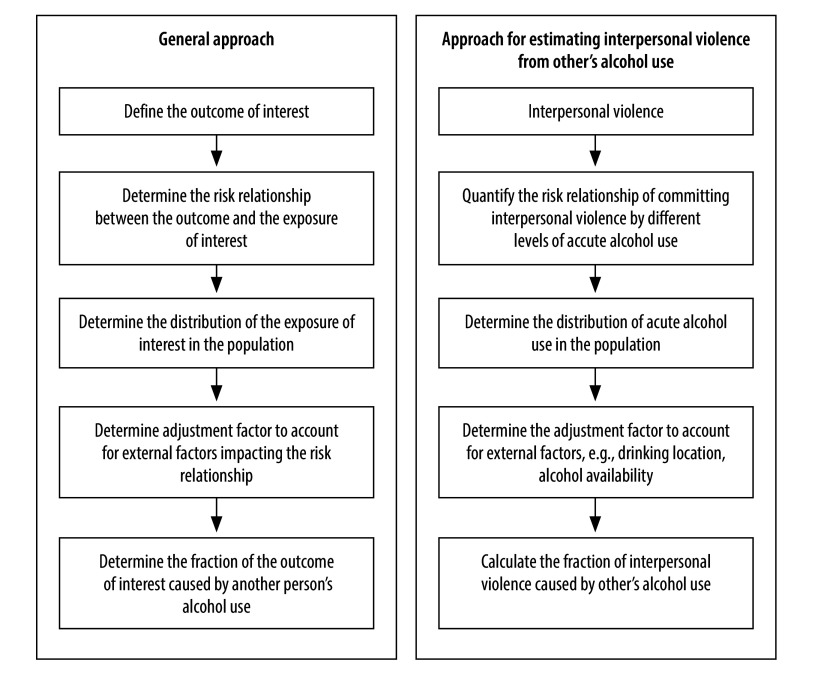
Indirect approach to estimate the health burden caused by others’ alcohol use

To overcome these challenges, we propose a research agenda to establish a consistent and evidence-based indirect approach. “Indirect” means that the share of events caused by alcohol use is not derived from one data source but is estimated by combining different data sources. This indirect approach is generally employed in the WHO burden of disease analysis and the GBD study, and can also be used to estimate the health burden of alcohol’s harm to others. We conceptually describe the necessary steps of the indirect approach in Fig. 1.

As a first step, the outcome of interest needs to be defined. As outlined above, alcohol’s harm to others covers a wide range of conditions from public disturbance to deaths due to traffic or intentional injuries. While all these conditions may result in physical and mental health consequences, they will contribute differently to the health burden as defined by premature deaths, years of life lost and disability-adjusted life years given their differences in severity. Burden of disease analyses therefore require a precise definition of the outcome of interest, including a specific set of conditions with reliably measurable health outcomes in line with common health classification systems. In the case of interpersonal violence, for example, an international standard is needed on which forms of violence are included (for example, emotional, physical and/or sexual violence) and whether the violence is directed against a specific group of people (for example, violence against children or intimate partners).

Next, the risk relationship between different doses of the exposure (alcohol use) and the outcome of interest needs to be understood. The shape of this relationship can take very different forms and describes the risk that the outcome (interpersonal violence) occurs with either (i) different levels of alcohol use (that is, perspective of the person doing the harm; Fig. 1); or (ii) different levels of someone else’s alcohol use (that is, perspective of the person experiencing the harm). For interpersonal violence, prior studies have primarily examined the link between acute alcohol use (such as blood alcohol concentration) and aggressive behaviour in research settings, which comes with obvious limitations in generalizability. Novel technologies can, however, help to minimize this limitation. For example, simulators and virtual reality can create artificial real-world experiences, improving the generalizability of laboratory studies, as has been proven in drink-driving research.^13^ Alternatively, researchers used ecological momentary assessment to study the association between situational alcohol use and intimate partner violence in couples with a history of partner aggression in real-life settings.^14^ More such research is needed to decipher the dose-dependent relationship between alcohol use and different alcohol’s harm to others outcomes in settings of high generalizability while preserving ethical principles.

Once the risk relationship is established, this information is combined with the exposure data, that is, (i) the distribution of alcohol use in the population; or (ii) the distribution of exposure to the alcohol use of others in the population. Given that most alcohol’s harm to others conditions are linked to acute alcohol use (rather than the average amount of alcohol consumed per day), we need to know how many people drink different levels of alcohol in specific situations. Doing so poses another challenge, as information on acute alcohol use is not readily available from surveys or population-level data sources. Previous studies have used data on heavy episodic drinking, such as drinking more than five drinks in one occasion, to approximate this information.^15^ Another option is to approximate acute alcohol use based on the distribution of the graduated quantity and frequency of alcohol consumed, as well as heavy episodic drinking, in the population. To the best of our knowledge, such a modelling exercise has not yet been undertaken.

A key challenge in quantifying alcohol’s harm to others is the role of external conditions that modify the risk of experiencing harms from others’ alcohol use. For example, alcohol use in public places, such as bars and pubs, may increase the likelihood of experiencing interpersonal violence from another person’s alcohol use; while living together with a partner and drinking alcohol at home may increase the risk of experiencing intimate partner violence. Such external factors may vary by sociodemographic factors, societies, and alcohol’s harm to other conditions, critically influencing the number of people at risk of experiencing specific harms. While they are not part of traditional burden of disease analysis, accounting for these external factors in the assessment of alcohol’s harm to others will be necessary. Doing so can be achieved by establishing an adjustment factor, determined by comparing burden of disease estimates derived from the direct and indirect approach. As a prerequisite, estimates from both approaches must be available. The adjustment factor is then determined based on the deviation of the indirect estimate from the direct estimate, assuming that the latter reflects the true distribution of alcohol’s harm to others in the population. While the adjustment factor merely quantifies a combination of external factors that are not further specified, complementary research, including qualitative, cohort or experimental studies, will contribute to decipher relevant external factors.

Finally, the fraction of interpersonal violence caused by another person’s alcohol use out of all recorded incidents of interpersonal violence can be calculated using the population attributable fraction approach accounting for the adjustment factor. This fraction can then be used to quantify the health burden attributable to interpersonal violence from others’ alcohol use by means of deaths or years of life lost upon the availability of reliable and valid data on the outcome of interest such as interpersonal violence.

## Policy implications

Upon progress in these pending steps, we will be able to describe the health burden caused by others’ alcohol use on a large scale that is yet insufficiently covered in global assessments of the burden of disease. A milestone for tobacco policy was the recognition of the carcinogenic properties of involuntary smoking by the International Agency for Research on Cancer in 2004^16^ – decades after this issue was first highlighted in the scientific literature.^17^ Acknowledging the evidence concerning second-hand harms clearly laid the foundation for integrating the obligation for protections from exposure to tobacco smoke in indoor workplaces, public transport, indoor public places and other public places into the WHO Framework Convention on Tobacco Control.^18^ After coming into force in 2005, this treaty has resulted in smoking bans on the grounds of protecting non-smokers in the years to come.^5^

Given the experience from tobacco policy, we expect alcohol’s harm to others to affect the policy debate. For example, we expect changes in the social acceptance of alcohol use in general and in public spaces in particular, as well as a strengthening of measures modifying the drinking environment and restricting the physical availability of alcoholic beverages. While these changes are welcome, we should actively combat an impending stigmatization of people with alcohol use disorders. Doing so involves the provision of adequate treatment, as opposed to the negligence or even criminalization of specific user groups such as women drinking alcohol during pregnancy,^19^ as well as significant efforts to improve alcohol and mental health literacy in the population. Eventually, succeeding in quantifying the entire health burden of alcohol could bring significant progress towards an international treaty on alcohol control analogous to that of tobacco.
